# Anticandidal Activity of a Siderophore from Marine Endophyte *Pseudomonas aeruginosa* Mgrv7

**DOI:** 10.3390/antibiotics13040347

**Published:** 2024-04-10

**Authors:** Essam Kotb, Amira H. Al-Abdalall, Ibtisam Ababutain, Nada F. AlAhmady, Sahar Aldossary, Eida Alkhaldi, Azzah I. Alghamdi, Hind A. S. Alzahrani, Mashael A. Almuhawish, Moudhi N. Alshammary, Asmaa A. Ahmed

**Affiliations:** 1 Department of Biology, College of Science, Imam Abdulrahman Bin Faisal University (IAU), P.O. Box 1982, Dammam 31441, Saudi Arabia; aalabdalall@iau.edu.sa (A.H.A.-A.); iababutain@iau.edu.sa (I.A.); nalahmadi@iau.edu.sa (N.F.A.); azalghamdi@iau.edu.sa (A.I.A.); malalmuhwish@iau.edu.sa (M.A.A.);; 2 Basic and Applied Scientific Research Center (BASRC), Imam Abdulrahman Bin Faisal University (IAU), P.O. Box 1982, Dammam 31441, Saudi Arabia; 3 Department of Statistics, Faculty of Commerce, Al-Azhar University, Cairo P.O. Box 11751, Egypt

**Keywords:** siderophore, antimicrobial, biocontrol, *Pseudomonas aeruginosa*, *Candida*, mangrove

## Abstract

An endophytic symbiont *P. aeruginosa*-producing anticandidal siderophore was recovered from mangrove leaves for the first time. Production was optimal in a succinate medium supplemented with 0.4% citric acid and 15 µM iron at pH 7 and 35 °C after 60 h of fermentation. UV spectra of the acidic preparation after purification with Amberlite XAD-4 resin gave a peak at 400 nm, while the neutralized form gave a peak at 360 nm. A prominent peak with RP-HPLC was obtained at RT 18.95 min, confirming its homogeneity. It was pH stable at 5.0–9.5 and thermally stable at elevated temperatures, which encourages the possibility of its application in extreme environments. The minimum inhibitory concentration (MIC) and minimum fungicidal concentration (MFC) against *Candida* spp. Were in the range of 128 µg/mL and lower. It enhanced the intracellular iron accumulation with 3.2–4.2-fold (as judged by atomic absorption spectrometry) with a subsequent increase in the intracellular antioxidative enzymes SOD and CAT. Furthermore, the malondialdehyde (MDA) concentration due to cellular lipid peroxidation increased to 3.8-fold and 7.3-fold in *C. albicans* and *C. tropicalis*, respectively. The scanning electron microscope (SEM) confirmed cellular damage in the form of roughness, malformation, and production of defensive exopolysaccharides and/or proteins after exposure to siderophore. In conclusion, this anticandidal siderophore may be a promising biocontrol, nonpolluting agent against waterborne pathogens and pathogens of the skin. It indirectly kills *Candida* spp. by ferroptosis and mediation of hyperaccumulation of iron rather than directly attacking the cell targets, which triggers the activation of antioxidative enzymes.

## 1. Introduction

*Candida* species are very pervasive organisms that can be found in food, digestive systems, mucocutaneous membranes, plants, and many other habitats worldwide. They can survive in both high-sugar and saline environments [1,2]. The widespread infection between individuals is common. In addition, the incidence of candidiasis after consumption of polluted water was also confirmed. The presence of pathogenic yeasts, such as *Candida albicans*, in river waters worldwide has raised many concerns about their effect on our health, especially in immunosuppressed persons [1,2,3]. *Candida* infections have become a global danger due to the appearance of unfamiliar clinical species and superbugs that obscure the treatment options. Their virulence is mainly due to the power to secrete lytic enzymes, adhere to epithelial cells, form biofilms, and transform from unicellular form to pseudomycelium form and vice versa. *C. albicans* and *C. tropicalis* are the first and second most virulent *Candida* species, respectively. *C. tropicalis* can invade the digestive system within only 30 min of internalization, which results in greater lethality [2].

Once inside, our immune system activates a signaling cascade in the yeast cells, which overproduces reactive oxygen species (ROS) that trigger the destruction of cell organelles and the killing of cells. This machinery of defense is very well studied in *C*. *albicans*, where researchers have discovered the *CAP1* gene, which is responsible for the production of counteracting antioxidative enzymes such as catalase (CAT), superoxide dismutase (SOD), glutathione S-transferase (GST), glutathione reductase (GR), and glutathione peroxidase (GPx) to repair the subcellular damage triggered by the oxidative stress molecules [4]. Fluconazole and echinocandin are the most common drugs used for the treatment of *Candida.* Infections that are resistant to drugs have very few treatment options; in addition, the alternative treatment option, amphotericin B, can be toxic for patients who are already very sick. This encourages the necessity for the innovation and production of novel helpful drugs and strategies. The bioactive materials from plants and microorganisms may be the finest alternatives [3,5].

Intertidal marine plants, predominantly mangrove trees, form a unique and dominating ecosystem on tropical coasts. Traditional medicine uses mangrove tree extracts to fight many microbial infections. Endophytic pseudomonads have seldom been studied; hence, they were chosen for this investigation. Endophytes can produce various bioactive materials, including metabolic and biosynthetic enzymes [3,5,6]. Common commercial medicinal products, such as podophyllotoxin and azadirachtin, are produced by endophytes. Endophytes help plants to grow and develop and make them more medicinal by secretion of growth regulators, phosphate solubilizing enzymes, deaminases, improving nitrogen fixation, and synthesis of antimicrobial substances and siderophores that stop colonization of attacking microbes and parasites. The variation in the environment in which the plants grow affects the endogenous ecosystem of endophytes and their metabolites [5,6,7].

*Pseudomonas* species belong to the endophytic bacteria that can produce potent biocontrol agents against pathogenic microorganisms. They can produce many secondary metabolites to protect plants from microbial infections [6]. Many other endophytic microorganisms were found to produce potent inhibitors against *Candida*; *Drechmeria* sp. from the roots of *Panax notoginseng* was found to produce novel drechmerins A-G; the MIC value of drechmerin B against *C. albicans* was 12.5 μg/mL [6]. Novel anticandidal polyketides were isolated from *Phoma* sp. SYSU-SK-7; the MIC values for colletotric A, 3-hydroxy-5-methoxy-2, 4, 6-trimethylbenzoic acid, and orsellinic acid against *C. albicans* were 3.27 μg/mL, 2.62 μg/mL, and 2.10 μg/mL, respectively [3]. Three new inhibitors were produced by a marine strain, *Stachybotrys chartarum*; the MIC value of atranone Q against *C. albicans* was 8 μg/mL [8]. Nine sesquiterpenes and three diterpenes were isolated from *Xylaria* sp. YM 311647; the highest activity against *C. albicans* had an MIC value of 16 μg/mL [5]. Biatriosporin D produced by *Biatriospora* spp. can suppress hyphal morphogenesis, biofilm formation, and adhesion of *C. albicans* [9]. In addition, the phytochemical carvacrol extracted from oregano (*Origanum vulgare*), thyme (*Thymus vulgaris*), and other aromatic plants inhibited *C. auris* at an MIC range of 125–500 µg/mL [10].

*Candida* and pseudomonads live in a socio-antagonistic interaction, and they can prevent themselves and other microorganisms from invasion and colonization by the production of biocontrol agents such as siderophores. The endophytic pseudomonads which will be used in this study as the source of an inhibitory siderophore are known to inhabit the interior of plants forming diverse microbial communities with unfamiliar metabolic capabilities enzymes [6,7,8]. The siderophores produced by endophytes are tiny structures that can capture iron from the surrounding environment when it is scarce. Multiple species of *Pseudomonas*, including *P. fluorescens*, *P. syringae*, *P. aeruginosa*, and *P. putida* can produce various forms of siderophores with many applications other than iron uptake, including their role as antimicrobial and/or permeabilizing agents for antibiotics and other drugs. This importance raises the need for further characterization and application of these promising agents [8,11,12]. The influential human pathogens, *C. albicans* and *P. aeruginosa*, can co-colonize or co-infect the same areas of the human body. Previous studies suggested mutual antagonistic interaction between them as a competition for the same shelter. The influence of mixed infections with both organisms on fungal and/or bacterial pathogenesis remains uncertain despite several clinical investigations documenting their occurrence [1,9,10].

To produce high amounts of siderophores, it is essential to study the nutritional and environmental factors affecting the producing bacterium. Production is based on the iron status and pH of the surrounding environment. The process is also driven by metal contaminants, temperature, nutrients, and other factors. Although the bioavailability of iron is strongly dependent on pH, siderophore production by different microbes appears to controvert the pH-dependency of iron deficiency. For example, acid soils are rich in hydroxamate siderophores produced mainly by fungi and *Streptomyces* and reflect in the optimal stability of their ferric complexes at low pH. In contrast, neutral to alkaline soils support the production of both catecholate and hydroxamate siderophores. Especially for catecholate siderophores, this condition is due to catecholates being designed for optimum iron binding at neutral conditions, with a tendency to lose ferric iron at low pH conditions. In *Bacillus* spp., siderophore production was greater between pHs 7.0 and 9.0 than at lower (5.0) and higher (11.0) pH. Therefore, the effect of pH on siderophore production is both species and structure-dependent [11]. The current research is the first investigation on pyoverdine (PVD) siderophore production from an endophytic halophilic strain of *P. aeruginosa* recovered from mangrove leaves. In addition, its antimicrobial potential against *Candida* spp. at the subcellular level was explored.

## 2. Results

### 2.1. Isolation and Screening for PVD-Producers

**Figure 1:**
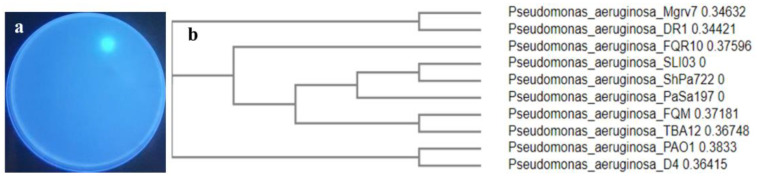
Showing the selected siderophore-producing isolate Mgrv7. Panel (**a**) represents its fluorescence under UV illumination (wavelength 365 nm) when grown on CAS agar of pH 7.0 for 48 h at 25 °C. Panel (**b**) represents the phylogenetic tree based on partial *16SrDNA* gene sequence (Accession number PP024541).

Out of ten endophytic pseudomonads recovered from mangrove leaves, isolate number 7 was selected as the most efficient siderophore producer on Chrome azurol sulfonate (CAS) agar (Figure 1a). The isolate was Gram-negative, motile, rod-shaped, and was able to grow in a cetrimide medium. It was positive for catalase, oxidase, citrate utilization, and l-arginine dehydrolase tests, while it was negative for indole, Voges Proskauer, and methyl red tests. Molecular characterization centered on the *16SrDNA* gene sequence was performed, and the strain showed almost 100% similarity with *P. aeruginosa* isolate DR1 (Sequence ID: LN889752.1). The BLAST search tool was applied to assess sequence homology and the sequence was blasted into the National Center for Biotechnology Information (NCBI) GenBank under accession number PP024541. The blasted tree was produced using ClustaW2-Phylogeny v2.1 available on European Bioinformatics Institute (EBI) tools; the Neighbour-joining method was applied (Figure 1b) and a strain code Mgrv7 was designated.

### 2.2. Optimization of Siderophore Productivity

**Figure 2:**
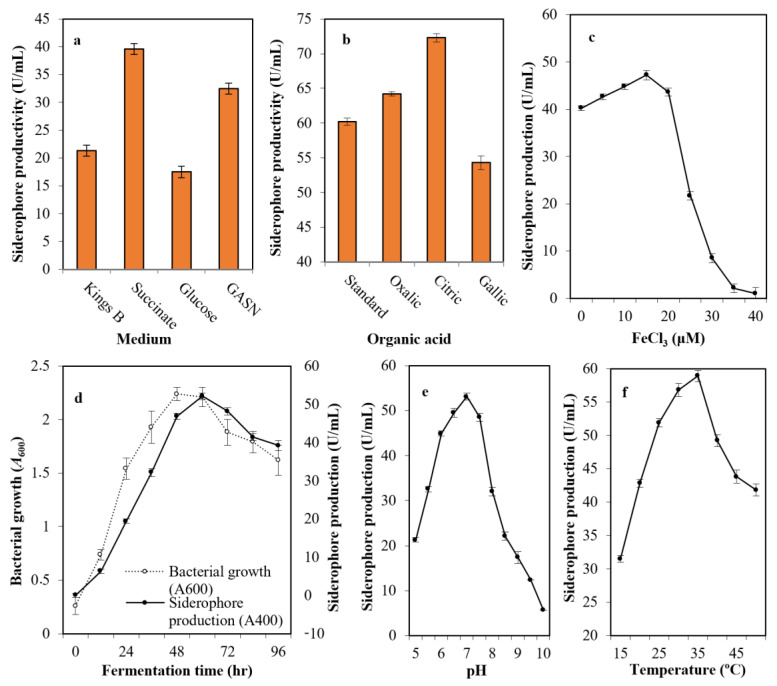
The optimized parameters influencing siderophore productivity; medium type (**a**), organic acid supplementation (**b**), iron concentration (**c**), growth phase (**d**), medium pH (**e**), and incubation temperature (**f**). The preliminary production of siderophore in liquid medium was performed by inoculation of two milliliters of 10^8^ colony forming units/mL Luria-Bertani (LB) broth in 25 mL of Kings B of pH 6.0 in a 100 mL volume Erlenmeyer flask. Incubation was conducted at 25 °C for 48 h at 120 rpm. The fermented broth was centrifuged at 5000 rpm for 20 min. Siderophores were estimated in cell-free supernatants; direct quantitative measurements were performed at wavelength 400 nm, with each unit quantified as the amount of siderophore producing an increase in absorbance of 0.1. Error bars represent the means ± standard deviations (n = 3). One-way analysis of variance (ANOVA) test indicated that the differences between means were significant since the *p*-values were lower than 0.5.

After growing the selected strain (Mgrv7) into the most common media cited in the literature—Kings B medium, succinate medium, GASN medium, and glucose medium—it was found that succinate medium (39.6 U/mL), followed by GASN (32.5 U/mL), was the best with a 1.86-fold increase when compared with Kings B (21.3 U/mL), and a 2.26-fold when compared with glucose medium(17.5 U/mL) (Figure 2a). The addition of citric acid (72.3 U/mL) and oxalic acid (64.2 U/mL) at 0.4% (*w*/*v*) to succinate medium improved siderophore productivity, while gallic acid did not (54.3 U/mL) (Figure 2b).

The impact of iron intensity in the culture medium was assessed and was found optimal at 15.0 μM concentration (47.2 U/mL). The productivity did not increase significantly at lower levels of iron, while the productivity was minimized drastically by increasing iron concentration. For this, 15 μM concentration was identified as the threshold value (Figure 2c). The production of siderophore fluctuated with time (0–96 h), and the maximum amount was observed after 60 h (52.1 U/mL) after bacterial growth peaked at 48 h of incubation (*A*_600_ = 2.24) (Figure 2d).

The pH of the production medium plays an influential function by causing physiological variations in cells and the transport of essential nutrients and extracellular enzymes across cell membranes. Figure 2e shows the ability of strain Mgrv7 to produce the siderophore at a wide pH range of 6.0 to 7.5, with a maximum at pH 7.0 (53.1 U/mL). Incubation temperature also plays an influential role in siderophore production. The bacterium was able to produce high levels of siderophore at a wide spectrum of temperatures (25–35 °C) with optimal levels at 35 °C (58.9 U/mL) (Figure 2f).

### 2.3. Properties of the Purified Siderophore

**Figure 3:**
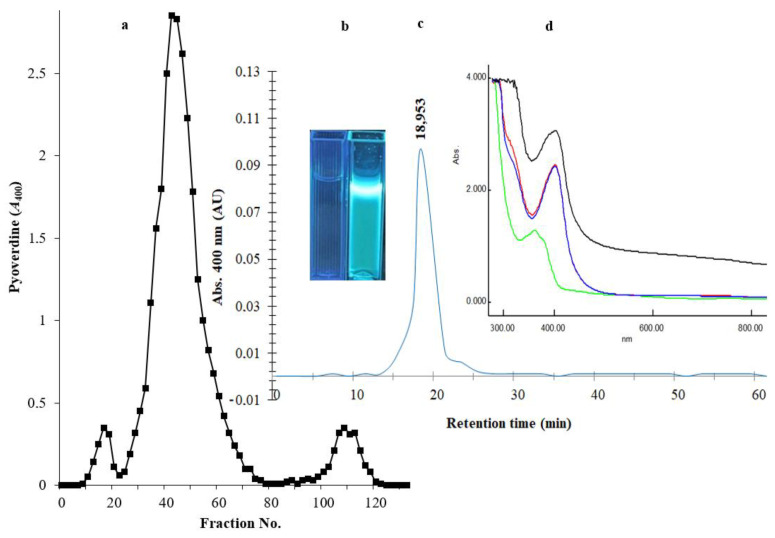
Panel (**a**) represents the chromatogram of PVD purification through an Amberlite XAD-4 column (2.5 × 20 cm^2^). The elution of immobilized PVD was performed with 50% (*v*/*v*) methanol at a speed of 5 mL/min. Panel (**b**) represents two cuvettes under a UV lamp of wavelength 365 nm; the left cuvette contains the elution solvent only, and the right cuvette contains the purified PVD. Panel (**c**) HPLC-visible chromatograms of the purified siderophore analyzed by reverse-phase HPLC using absorbance determination at wavelength 400 nm. Panel (**d**) represents the UV spectra of the 0.2 µm filtrate (black line), the chloroform extraction layer (red line), the acidified form of PVD at pH 3.0 (blue line), and the neutralized form of PVD at pH 7.0 (green line).

After the removal of bacterial cells from culture broth by centrifugation and the removal of macromolecules by a 0.2 µm membrane, an equal amount of chloroform was mixed with the filtrate for the extraction of PVD. The PVD/chloroform layer was separated, and the chloroform evaporated with a Hei-VAP Heidolph rotary evaporator. The recovered PVD was suspended in water at 10 mg/mL concentration and applied onto an Amberlite XAD-4 column (2.5 × 20 cm^2^). A major homogenous peak consisting of fractions between numbers 26 and 68 was recovered by elution with 86 mL of 50% (*v*/*v*) methanol (Figure 3a). The fluorescence of siderophore-containing fractions was confirmed under a UV illumination lamp at a wavelength of 365 nm (Figure 3b). To investigate the type of PVD purified from *P. aeruginosa* Mgrv7, positive fractions were analyzed by RP-HPLC. A prominent peak was obtained at an RT of 18.953 min, confirming its homogeneity (Figure 3c). The characteristic green color of PVD was lost at pH 3.0 and was retained after neutralization by NaOH. The UV spectra at the wavelength range of 300–900 nm showed maximum absorption at wavelength 400 nm and a small peak at ~320 nm after the 0.2 µm filtration, chloroform extraction, and acidification to pH 3.0, while it gave a peak at 360 nm when re-neutralized to pH 7.0 (Figure 3d).

### 2.4. Thermal Stability and pH Stability of Siderophore

**Figure 4:**
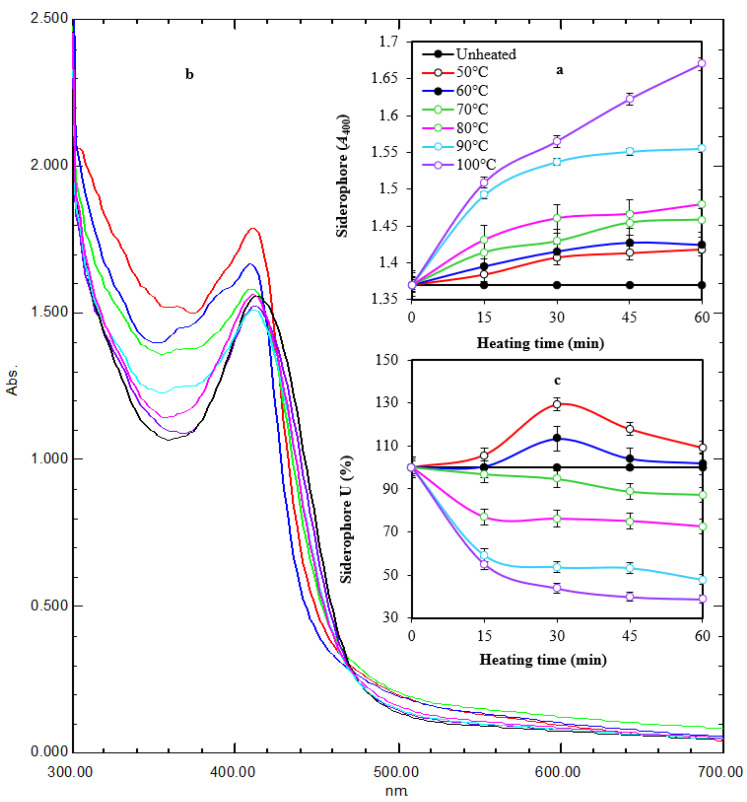
Time course of thermal treatment of tested siderophore at different temperatures for 60 min (**a**). Heating changed the characteristic color and fluorescence of siderophore gradually with maximum change at 100 °C after 60 min of exposure. UV spectra of heated preparations after 1 h of exposure (**b**). Heating changed the absorption peak from 414 nm to 410–412 nm. In addition, the optical density increased in comparison with the non-heated preparation. The reactivity of treated preparations at *A*_630_ followed a regression pattern at 70–100 °C and a stimulative effect at 50–60 °C (**c**). Error bars represent means ± standard deviations (n = 3). Some lines had no error bars because the value was 100%. Two-way ANOVA for panel a revealed that both temperature and time had effects on siderophore in form of *A*_400_, as the *p*-values were 0.0002 and 0.000003, respectively. This agrees with the values of correlation coefficient between the time and *A*_400_ under different temperatures, which were between 0.88 and 0.97, meaning that it was a very strong positive correlation. The correlation between temperature and *A*_400_ under different times was between 0.89 and 0.92, meaning that it was a very strong positive correlation. In panel (**c**), two-way ANOVA revealed that temperature had an effect on siderophore in the form of *A*_630_ as the *p*-value was 0.0000009, while there was insufficient evidence to conclude that *A*_630_ depends on the time since the *p*-value was 0.06 > 0.05. These ensure a correlation between the temperature and *A*_630_ under different time values where the correlation coefficient was between −0.66 and −0.76, which means that there was a strong negative correlation. The relation between time and *A*_630_ seemed to be unstable since for lower temperatures, the correlation between time and *A*_630_ was a weak positive correlation (between 0.2 and 0.4), while for higher temperatures, it was a very strong negative correlation (between −0.80 and −0.99).

**Figure 5:**
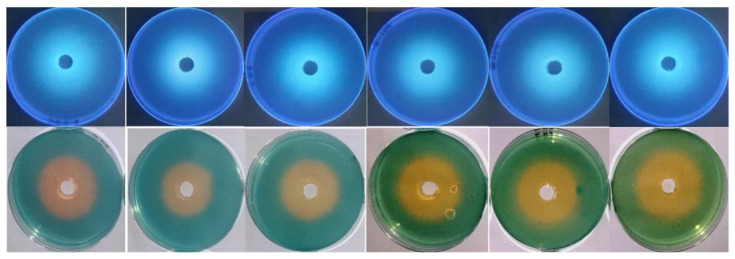
pH stability of siderophore at pH values 5.0, 6.0, 7.0, 8.0, 9.0, and 9.5 from left to right direction. Siderophore preparations were adjusted to these pH values, and CAS agars were prepared at the same range. Each well was supplied with 0.5 mL of siderophore preparation. Incubation was conducted at 35 °C for 24 h. The appearance of orange halos on CAS agars and their fluorescence under UV were taken as indicative of their remaining activity and stability at the tested pH values.

It appeared that thermal treatment (50–100 °C for 60 min) of siderophore slightly increased its optical density linearly at 400 nm by increasing both temperature and treatment time (Figure 4a). Maximum absorption (*A*_400_ = 1.670) was obtained after exposure at 100 °C for 60 min, while lowest absorption (*A*_400_ = 1.384) was obtained after exposure at 50 °C for 15 min compared with *A*_400_ of 1.370 for non-heated samples. Scanning at 300–700 nm for thermally treated preparations after 1 h of exposure showed that the maximum absorption peak was slightly shifted from 414 nm to 410–412 nm. In addition, the optical density increased in comparison with the non-heated preparation. The optical density increased to 1.035–1.219-fold at 50–100 °C, respectively (Figure 4b). In parallel, the reactivity of treated preparations in the form of siderophore units (%) was assessed by the spectrophotometric CAS assay at wavelength 630 nm (Figure 4c). The remaining units were 87.3%, 72.5%, 47.8%, and 38.8% after 60 min of exposure at 70–100 °C, respectively, while the remaining units after exposure to 50 °C and 60 °C for 60 min were 109.1% and 101.8%, respectively, indicating a stimulative effect, especially after 30 min of exposure. Interestingly, siderophore preparations showed remarkable pH stability in a wide pH range (5.0–9.5) for 24 h, as reflected by the appearance of orange coloration on CAS agars and their fluorescence under UV (Figure 5).

### 2.5. Anticandidal Activity of Siderophore and SEM Examination

During studying the effects of the siderophore from *P. aeruginosa* Mgrv7 against various pathogenic Candida spp. variable killing and/or inhibitory degrees were observed (Table 1). The best effect for PVD was observed against *C. albicans* ATCC 14053 and *C. tropicalis* ATCC 13803, respectively. The inhibition zone against *C. albicans* ATCC 14053 was 19.5 mm with MIC of 16 µg/mL and MFC of 16 µg/mL. In addition, the inhibition zone against *C. tropicalis* ATCC 13803 was 17.2 mm with MIC of 16 µg/mL and MFC of 32 µg/mL. The effect of siderophore was lower against the other species of Candida, as shown in Table 1. The lowest effect was against *C. glabrata* ATCC 15126 and *C. auris* ATCC MYA-5001; the MIC was 128 µg/mL and 64 µg/mL. In addition, the MFC was >128 µg/mL and 128 µg/mL, respectively. The tolerance levels in the form of MFC/MIC for all tested species were between 1.0 and 2.0, which indicated a killing effect for tested PVD as it was less than 4.0.

**Figure 6:**
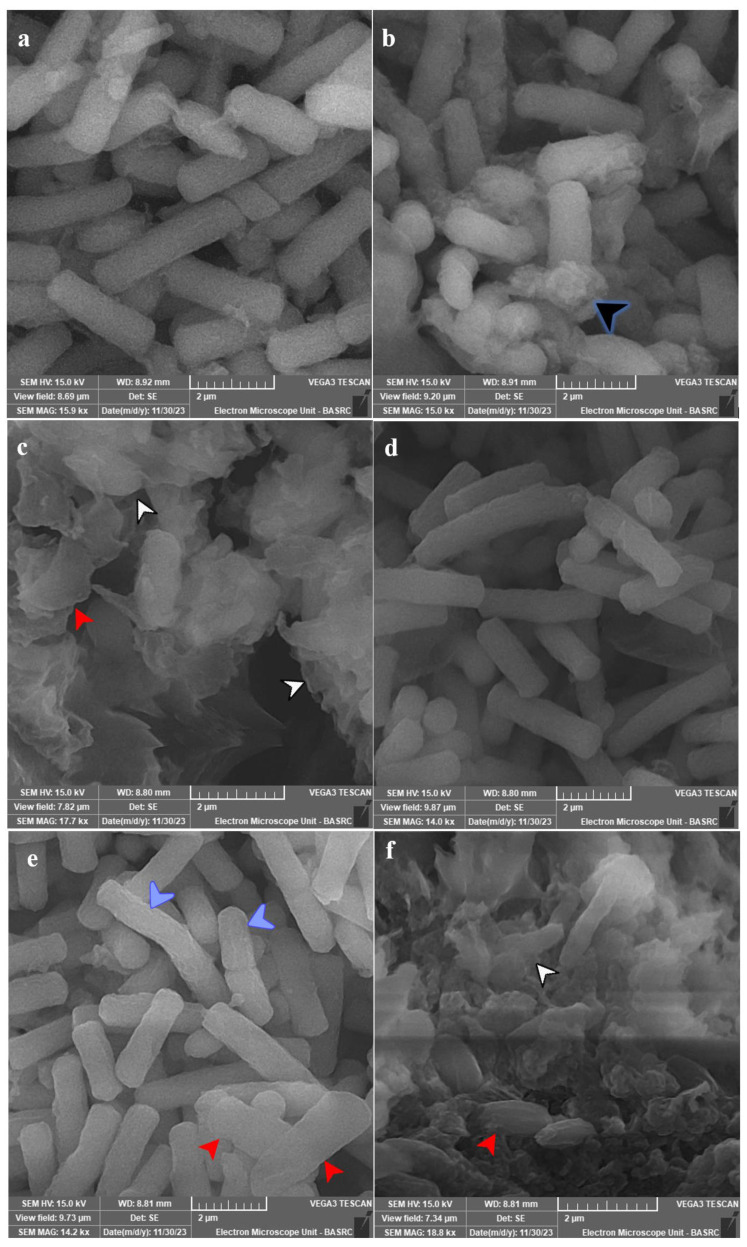
SEM micrographs for tested species of Candida. Panel (**a**) represents untreated *C. tropicalis* ATCC 13803. Panels (**b**,**c**) show the treated cells with 10 µg PVD/mL after 12 and 24 h. Panel (**d**) represents the untreated cells of *C. albicans* ATCC 14053. Panels (**e**,**f**) show the treated cells with 10 µg PVD/mL after 12 and 24 h. Growth was allowed in Yeast Extract Peptone-Dextrose (YPD) broth for 24 h at 37 °C and 120 rpm shaking speed. *C. tropicalis* showed production of exopolysaccharides and/or proteins (black arrowhead) after 12 h of exposure, which were massive with cellular lysis (white arrowhead) and loss of shape (red arrowhead) after 24 h of exposure. *C. albicans* showed more surface roughness (blue arrowhead) after 12 h of exposure, which was more obvious after 24 h of exposure with loss of shape (red arrowhead) and cellular lysis (white arrowhead).

SEM analysis for most affected species was conducted, and the subcellular examination of treated *C. tropicalis* ATCC 13803 showed the formation of exopolysaccharides and/or proteins after 12 h of exposure, which were massive with cellular lysis and loss of shape after 24 h of exposure (Figure 6a–c). *C. albicans* ATCC 14053 showed more surface roughness after 12 h of exposure, which was more obvious after 24 h of exposure with loss of shape and cellular lysis (Figure 6d–f).

### 2.6. Intracellular Iron Content

**Figure 7:**
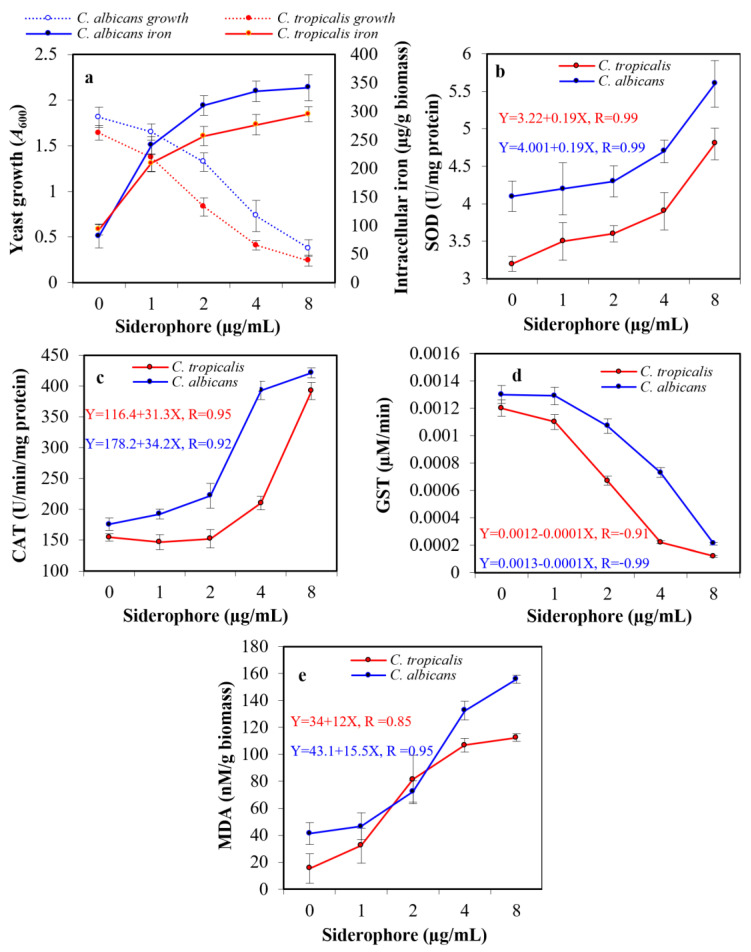
Effect of the subinhibitory concentrations of siderophore on the growth and intracellular iron accumulation in *C. albicans* and *C. tropicalis* (**a**). In addition, the ROS accumulation was indicated by direct assessment of the activity of intracellular antioxidative enzymes, superoxide dismutase (SOD, (**b**)), catalase (CAT, (**c**)), glutathione S-transferase (GST, (**d**)), and lipid peroxidation by release of malondialdehyde (MDA) from the cellular organelles and contents (**e**). The statistical study for panel a proved that increased iron internalization had a negative effect on the growth of *C. albicans* and *C. tropicalis*, as the correlation coefficients were −0.81 and −0.92, respectively. The effect upon *C. tropicalis* was higher, as the increase in siderophore concentration led to a greater increase in iron internalization since the concentration of siderophore had a positive correlation with iron accumulation (0.73 and 0.78, respectively). The relation between SOD, CAT, GST, MDA, and the growth of both Candida were studied in the form of linear regression (equations are represented in figures).

The exposure of pathogenic yeasts to tested siderophores is supposed to change their cellular iron level. To judge whether the observed growth inhibition in the previous experiment affected by the extracellular iron chelation with siderophore, making iron unavailable or randomly internalized into the tested cells, an assay for intracellular iron was created. When yeast cultures were exposed to siderophore in the presence of iron available in medium components, growth was suppressed by increasing siderophore concentration, while intracellular iron accumulation increased within both cell types. In the presence of 8 µg siderophore/mL, intracellular iron content in the case of *C. albicans* was 341.8 μg/g biomass, which was 4.2-fold higher than that of the blank treatment (81.4 μg/g biomass). In the case of *C. tropicalis*, the internal iron concentration reached 295.4 μg/g biomass, which was 3.2-fold higher than that of blank (93.2 μg/g biomass). Therefore, the addition of siderophore caused a significant increase in the intracellular iron in both organisms (Figure 7a).

### 2.7. Assessment of Antioxidative Enzymes

Assessment of the probable oxidative stress in siderophore-treated yeast cultures was performed by evaluating the enzymatic activity of the antioxidative enzymes SOD, CAT, and GST. Additionally, the release of MDA due to peroxidation membrane lipids was evaluated. No substantial difference was detected in the enzymatic activity of both SOD and CAT after treatment of both yeasts with siderophore at 1–2 μg/mL concentration. However, a significant change in SOD was detected at 8 μg/mL with 1.4-fold and 1.5-fold increase when evaluated against the blank treatments of both *C. albicans* and *C. tropicalis*, respectively (Figure 7b). In addition, an elevation in CAT was detected at 8 μg/mL with 2.4-fold and 2.5-fold increase when evaluated against the blank treatments of both *C. albicans* and *C. tropicalis*, respectively. It was noticed that CAT activity in the case of *C. tropicalis* upon treatment with 1–2 μg/mL was lower than that of the control (Figure 7c). On the other hand, GST enzymatic activity showed a significant decrease of 6.2-fold and 10.0-fold at 8 μg/mL as compared to control in *C. albicans* and *C. tropicalis*, respectively (Figure 7d). Maximum MDA concentration was found at 8 μg siderophore/mL as compared to controls, with a significant increase of 3.8-fold and 7.3-fold for *C. albicans* and *C. tropicalis*, respectively (Figure 7e).

## 3. Discussion

Although siderophores are mainly secreted by bacteria for the acquisition of iron molecules from the outer environment, they found other applications such as plant growth promotions, environmental applications, biocontrol of pathogenic bacteria, yeasts, and molds, and other medical applications [7,11,12]. Several reports demonstrated the capability of the genus *Pseudomonas* to produce extracellular siderophores. However, detailed characterization and antimicrobial activity at the subcellular level were not fully performed. In the current research, ten endophytic pseudomonads were recovered from plant leaves of a mangrove forest located in the coastal region of Tarut Island, Saudi Arabia, for the first time. Based on the screening program, the maximum amount of siderophore was secreted by isolate number 7, which was characterized biochemically and molecularly as *P. aeruginosa* and given Mgrv7 as a strain code and PP024541 as an accession number in the GenBank database. Mangroves have several endophytic symbionts that generate powerful antibacterial siderophores, according to this research. This work also implies that endophytes from tough and competitive habitats like mangrove ecosystems may provide novel infection-fighting drugs.

To produce high amounts of siderophore, the physical and nutritional factors affecting its biosynthesis and productivity by strain Mgrv7 were optimized. These results are very important for other researchers and industrialists to upstream and scale up the production process to increase the economic benefits and performance of the system, improve the process or models, and can also help to reduce costs, increase efficiency, and improve the quality of the results. The optimal productivity was found in the case of succinate medium in agreement with *P. aeruginosa* marine isolate [12], *P. fluorescens* strain P5-18 [13], and *Pseudomonas* strains PRS and GRP3A [14]. However, Kings B was optimal for the induction of siderophore productivity from *P. aeruginosa* strain ryn32 [15], and GASN was superior for *Pseudomonas* sp. ANT_H12B [16].

The production of siderophore by strain Mgrv7 increased to 1.2-fold after the addition of citric acid. This might be because our strain could assimilate citric acid easily out of all tested organic acids. The enhancement of siderophore production from *P. aeruginosa* was also reported when the culture medium was supplemented with citric acid [17]. However, citric acid was not preferred for producing siderophores from *P. fluorescence* and *P. putida* [18].

It was found that trace concentrations of iron to 15.0 μM were optimal for siderophore productivity from strain Mgrv7. Concentrations above this threshold level inhibited siderophore production gradually. This might be attributed to the negative transcriptional control by fur protein where ferrous iron (Fe^+2^) functions as a co-repressor for the producing organism. These lower levels of iron were also confirmed for siderophore production from *P. aeruginosa* FP6 (5 µM) [19], *P. aeruginosa* strain ryn32 (25 µM) [15], *Pseudomonas* strain GRP3A [14], and three *P. fluorescens* isolates (0 µM) [20].

The highest level of siderophore productivity by strain Mgrv7 was observed at 60 h after its growth peaked at 48 h of incubation. The maximum productivity was exactly when the bacterial growth was in the death phase. This explains a reciprocal relation between siderophore productivity and bacterial growth, a characteristic of secondary metabolites. This resembles siderophore production by *P. aeruginosa* ryn32 (60 h) [15], while it is different from *Pseudomonas* strains PRS and GRP3A [14].

The highest level of productivity was found at 25–40 °C with a peak at 35 °C. This result is comparable with *P. aeruginosa* ryn32 (35 °C) [15] and *P. aeruginosa* (27.8 °C) [21]. The optimal pH for siderophore production by strain Mgrv7 was found at a range of 6.0–7.5, with a peak at pH 7.0. This may be significant in the natural habitat of such bacteria because iron is found in an insoluble form at pH 6.5 and is not obtainable by cells. This stress of iron induces siderophore production. This is close to other investigators, where pH 6.0 was optimal for a marine strain of *P. aeruginosa* [12]. pH 7 was optimal for *P. putida* NCIM 2847, *P. aeruginosa* FP6 [19], and *P. aeruginosa* [21]. In addition, pH 7.5 was best for *P. aeruginosa* ryn32 [15]. The broader range of pH (5–8) for productivity was also observed for *Pseudomonas* sp. ANT_H12B [16]. The effect of medium pH on siderophore productivity may be due to the ability of free protons (H^+^) to induce physiological changes in the bacterial cells, siderophore biosynthesis, and the transport of several nutrients across the cell membranes.

After purification of siderophore from strain Mgrv7 by Amberlite XAD-4 resin column chromatography, the UV absorption spectra were assessed for both the acidic preparation and the neutral preparation. The spectral variation between siderophore preparations at pH 3.0 and 7.0 was obvious; no charge transfer bands were found at *A*_400_ for the acidic preparation, and PVD completely lost its characteristic color. The spectrum of the acidic preparation peaked at wavelength 400 nm while the neutral preparation peaked at ~360 nm. Comparable results were obtained for the neutral chloroform extract of PVD from *P. aeruginosa* (362 nm) [22], PVDs of *P. aeruginosa* 4EA (405 nm and 370 nm) [23], and *P. aeruginosa* strains PAO1 (400 nm) [24].

The final purified PVD was subject to HPLC with a C18 reverse phase column to verify its purity. In addition, the RT determination from HPLC analysis allows the differentiation between typical and atypical PVDs of *Pseudomonas* spp. [25]. One major peak at RT of 18.95 min was obtained, which is indicative of the purity and homogeneity of PVD-Mgrv7. This RT is close to those found in the atypical PVDs Pa B (19.07 min, ΔRT 0.12 min), Pa A (18.78 min, ΔRT 0.17 min), and the PVD of a *Pseudomonas* sp. isolated from Chickpea rhizosphere (18.44 min, ΔRT 0.51 min) [26]. However, the retention times of the atypical PVD Pa C (28.04 min, ΔRT 9.09 min), typical PVD-PAO1 (22.5 min, ΔRT 3.55 min), PVD of *P. fuscovaginae* UPB 1023 (22.10 min, ΔRT 3.15 min), and the PVD of a strain of *P. aeruginosa* (22.6 min, ΔRT 3.65 min) were different [25]. The results of UV spectra and the visual change in color of PVD observed at pH 7.0 and 3.0 collectively indicated that PVD-Mgrv7 is an atypical PVD type [27,28].

To withstand harsh environmental conditions, thermally stable siderophores are particularly important. Interestingly, the thermal treatment of the siderophore increased its optical density (*A*_400_) linearly by increasing both temperature and treatment time. Furthermore, the spectrophotometric scanning at wavelengths 300–700 nm showed a slight shift in the maximum absorption peak from 414 nm to 410–412 nm, and the *A*_400_ increased to 1.035–1.219-fold at 50–100 °C. These slight changes due to the thermal treatment might be due to a minor change in the characteristic PVD chromophore or its short peptide, leading to different absorption characteristics due to the charge transfer. Comparable heat stability and structural consistency of these cyclic siderophores have been reported in earlier reports [29]. Interestingly, although the tested siderophore lost its characteristic color at acidic pH values, all preparations were stable at the full range of tested pHs (pH 5.0–9.5). This encourages the possibility of its application in various acidic and alkaline environments.

Previous studies suggested antagonistic communications between *C. albicans* and *P. aeruginosa* during infection and that *P. aeruginosa* affects surface structures and inhibits the dimorphism of *C. albicans*. However, the impact on fungal pathogenesis is still unclear [18]. During the study of the effect of PVD-Mgrv7 siderophore against *Candida* spp., variable inhibitory degrees were observed. The MIC, MFC, and tolerance level (ratio MFC/MIC) were assessed. The tested siderophore could be considered a strong anticandidal agent, as it scored excellent MIC and fungicidal values (16–128 µg/mL). The calculated MFC/MIC value was between 1 and 2; it is recognized in the previous studies that an antimicrobial substance is fungicidal, not fungistatic, when the value of MFC/MIC ≤ 4 [30]. Referring to other investigators, MFC for catecholate siderophore from *E. coli* against *Aspergillus nidulans* was 54 µg/mL with malformation of fungal morphology at lower concentrations [6]. The MFC of a pyocyanin siderophore from *P. aeruginosa* against *C. albicans* and *Aspergillus fumigatus* were ≥100 µg/mL, and the lower levels inhibited yeast transformation [31]. In addition, a siderophore from *Brevibacillus brevis* GZDF3 has shown synergistic activity with amphotericin B against *C. albicans* [32]. Many reports suggested that siderophores produced by *P. fluorescens* MPF47 have potent biocontrol activity against *R. solani* [33]. Moreover, it was found that *P. syringae* BAF.1 siderophore demonstrated obvious antagonistic activity against *F. oxysporum* [34]. Interestingly, the bacillibactin siderophore produced by SQR9 was upregulated when it was confronted with fungi [35]. The siderophores of *P. fluorescens* BBc6R8 inhibited the growth of the actinomycete *Streptomyces ambofaciens* ATCC23877 [11]. The siderophores of *P. putida* had a greater antagonistic activity towards fungi [36]. Siderophore produced by *Alcaligenes feacalis* showed suppressive activity against *Fusarium oxysporum* NCIM1008 [37]. All these findings demonstrate the potential of siderophores as antifungal agents [11]. The importance of siderophore and ergosterol biosynthetic pathways for fungal virulence and antifungal treatment was confirmed, as iron starvation down-regulates the cellular ergosterol content but upregulates siderophore TAFC production by *A. fumigatus* [38].

Enhanced internalization of iron molecules was observed in both *C. albicans* and *C. tropicalis*, and their growth was inhibited by exposure to increasing concentrations of PVD-Mgrv7. In addition, oxidative stress in siderophore-treated cells, as reflected by the synthesis of the antioxidative enzymes CAT, SOD, and GST, was observed. This increase in intracellular iron concentration, as indicated by the atomic absorption spectrometry, mostly correlated with the production of ROS such as hydrogen peroxide, which, in turn, generates hydroxyl radicals (OH^•^) and hydroxyl ions (OH^−^) by Fenton reaction, causing further oxidative stress; where many studies reported that stresses including metal stress can shift the equilibrium of ROS homeostasis. To counteract this effect, microorganisms, including yeasts, settle a defensive mechanism, including the synthesis of the oxidative enzyme SOD that could break superoxides into H_2_O_2_, which is further hydrolyzed into H_2_O and O_2_ by the synthesis of other oxidative enzymes such CAT and GST [4,6]. However, the overproduction of ROS by both *Candida* cells adversely affected their morphological structures because it mostly led to the peroxidation of membrane lipids. The increase in MDA content due to lipid peroxidation at 8 μg siderophore/mL supports this opinion (this was confirmed in the next SEM study).

The increase in CAT and SOD enzymatic activity coupled with the reduction in GST activity after PVD exposure appears to be an effort to mitigate the presence of high amount of ROS, especially H_2_O_2_. This antioxidative response, however, seemed inefficient in restoring the normal physiology and status of treated cells because the ROS level was above their threshold. One of the major outcomes of intensified ROS production is membrane interruption, which results in loss of consistency. Therefore, growth suppression and mortality can be the result of uncontrolled oxidative stress and greater destruction of membranes. This damage has been correlated with ferroptosis, which arises only when iron is internalized, resulting in ROS production and lipid peroxidation, initiating cell death [33,39].

Concerning previous studies, a comparable rise in CAT, SOD, and MDA with a decline in GST activity has been detected in many fungi upon metal stress [40]. It was found that a copper- and zinc-containing SOD (Cu/ZnSOD) is essential for *C. albicans* to counteract the negative effects of various oxidative stresses. The ROS are recognized to deactivate the [4Fe-4S] cluster-containing enzymes in *Candida* spp. through the oxidation and release of iron from the cluster. The released iron can crosslink with H_2_O_2_ to produce OH^−^ radicals [1,6]. The OH^•^ and H_2_O_2_ radicals, in turn, can oxidize the cellular macromolecules, including proteins, lipids, DNA, and RNA, which results in the inhibition of enzymes, disruption of membranes, and generation of mutations, respectively [40]. In addition, it was established that intracellular concentrations of metals in yeasts are primarily regulated by sequestration with the cellular glutathione molecules. For this, metals are assumed to commence oxidative stress either by the reduction in glutathione concentration or by the displacement of Zn^2+^ and Fe^2+^ ions from proteins and enzymes [1]. Thus, there may be a connection between the transcriptional responses of *C. albicans* to oxidative stresses and metal stresses.

The quantities of the secondary antioxidative enzyme GST displayed an average decrease of 6.2–10.0-fold when exposed to 8 μg siderophore/mL. This decrease might be attributed to the ability of the siderophore to bind the fatty acids constituting the GST [41]. This coincides with former studies about antifungal agents against *Candida* species [10].

The current study emphasizes that the exposure of yeast cells to xenosiderophore assists in improved uptake of iron, perhaps via MirA receptors, which, in turn, induces uncontrolled hyperaccumulation of iron, causing ferroptotic death in *Candida* spp., the same as *A. nidulans* [6]. This opens the direction for investigating the effectiveness of siderophores against other pathogenic microorganisms. Fungi such as *Magnaporthe oryzae* were also reported to experience ferroptotic cell death [42]; however, to the best of our knowledge, there are no previous reports about ferroptosis in *Candida* spp. On the other hand, some reports attributed the surface changes in treated cells after the exposure to siderophores to the cellular depletion of free iron and other metals due to complexation with siderophores outside cells and/or coupling of wall cations with siderophores [43]. This creates an iron-starved condition in the absence of any compensatory mechanism. This iron-blockage strategy might disrupt the transmembrane potential and ATP generation in microbial cells because iron is a key electron acceptor in the oxidative phosphorylation process that generates ATP molecules, which are required for all cellular functions and the keeping of transmembrane potential [44].

In this report, ultracellular examination of *C. tropicalis* cells treated with PVD-Mgrv7 siderophore by SEM showed the formation of exopolysaccharides and/or proteins after 12 h of exposure, which were massive with cellular lysis and loss of shape after 24 h of exposure. In the case of *C. albicans*, more surface roughness was observed after 12 h of exposure, which was more obvious after 24 h of exposure with loss of shape and cellular lysis. The massive exopolysaccharides and/or proteins produced by *C. tropicalis* cells may be a cellular shield against typical antifungals and may also be an explanation for reduced susceptibility to the siderophore. Moreover, this may be the explanation for higher MFC values when contrasted with *C. albicans* (32 µg/mL vs. 16 µg/mL, respectively). This indicates the ability of PVD-Mgrv7 to disrupt the cell wall and membrane and induce a physiological response by induction of oxidative stress. This is particularly important as both impacts are fundamental virulence tools in *Candida* spp. [1,32]. Regarding the subcellular impact of siderophores in other studies, a distortion in the morphology of phialides in *A. nidulans* was reported with increasing catecholate siderophore exposure [6]. Oxidative stress-induced morphological alterations were also reported in *A. niger* UCP/WFCC 126 [45] and *A. nidulans* [46] treated with different antifungal agents. Such studies also suggested the ability of oxidative stress induced by siderophore-mediated iron accumulation to adversely affect the topological features in microorganisms.

## 4. Materials and Methods

### 4.1. Chemicals and Reagents

CAS, hexadecyl trimethyl ammonium bromide (HDTMA), 1-chloro-2,4-dinitrobenzene (CDNB), glutathione, cetrimide agar, nutrient agar, nutrient broth, and LB broth obtained from Sigma-Aldrich (Burlington, VT, USA). Supplementary items for analysis were obtained from local suppliers. The media used in this investigation was sterilized by autoclaving at 121 °C for 15 min, with an air pressure of 15 psi, unless otherwise specified. CAS reagent was freshly prepared by combining two solutions. The first one was prepared by the addition of 60.5 mg of CAS to 50.0 mL distilled water and 10.0 mL solution containing 1.0 mM FeCl_3_·6H_2_O in 10.0 mM HCl. The second solution was prepared by combining 72.9 mg of HDTMA with 40.0 mL of deionized water. Both solutions were stored individually on the stirrer for 20 min. Subsequently, the second solution is gradually introduced into the first solution while continuously stirring. The pH of 100.0 mL CAS reagent was adjusted to 7.0 (pH meter, Mettler Toledo, Greifensee, Switzerland). Ultimately, a solution with an intense azure was generated and sterilized. CAS agar was made by the addition of 100.0 mL of sterile CAS reagent to 900.0 mL of sterile nutrient agar of pH 7.0.

### 4.2. Isolation of Endophytic Pseudomonads from Mangrove Forest

Healthy plant leaves were collected from the mangrove forest of Tarut Island, Saudi Arabia (26.598634348372382, 50.063778644367034). The method used [47] to recover the endophytic pseudomonads was simply to remove dirt and then leaves washed in sterile water. Surface sterilization was made by immersion in 70% ethyl alcohol for 30 s and 5% sodium hypochlorite for 1 min. Mangrove leaves were then rinsed with sterile distilled water. Each gram of leaves was pulverized in a sterile mortar with 9 mL of phosphate-buffered saline (PBS). The suspension was decimally diluted in physiological saline till 1:1000 dilution. To cultivate endophytic pseudomonads, one milliliter from each dilution was plated onto cetrimide agar at 25 °C for 2 days. Quadrate streaking on the same medium served to purify the obtained isolates. The isolates were then kept on cetrimide slants at 4 °C and in 15% (*v*/*v*) glycerated LB broth at −80 °C.

### 4.3. Characterization of the Selected Isolate

Isolate Mgrv7, which showed the most efficient productivity of siderophore, was identified and characterized biochemically based on the growth in cetrimide medium, catalase test, oxidase test, indole test, citrate utilization test, methyl red test, Voges Proskauer test, and l-arginine dihydrolase test. Identification was then confirmed molecularly by forward and reverse DNA sequencing reaction of PCR amplicons with *16SrDNA* gene. The DNA was extracted and the *16SrDNA* gene was amplified with PCR using 1492R primer (5′-CGG (CT) TACCTTGTTACGACTT-3′) and 27F primer (5′-AGAGTTTGATC (AC) TGGCTCAG-3′). Agarose gel electrophoresis and DNA sequencing were followed. Then, the sequence was searched for homology using the Basic Local Alignment Search Tool (BLAST) from the GenBank library (http://www.ncbi.nlm.nih.gov, accessed on 27 December 2023).

### 4.4. Production and Assay of Siderophore

The initial qualitative detection of siderophore productivity was performed by spotting the recovered pseudomonads on CAS agar plates (pH 7.0) and incubating them under dark conditions at 25 °C for 48 h. The formation of orange zones was indicative of siderophore productivity. For preliminary production of siderophore in the liquid medium, two milliliters of 10^8^ colony forming units/mL LB broth were used to inoculate 25 mL of Kings B of pH 6.0 in a 100 mL volume Erlenmeyer flask. Incubation was performed at 25 °C for 48 h at 120 rpm. The fermented broth was centrifuged at 5000 rpm for 20 min. Siderophores were measured in cell-free supernatants following centrifugation at 5000 rpm for 15 min (Centrifuge REF: 1406, Hettich, Tuttlingen, Germany).

Unless otherwise mentioned, direct quantitative measurements of fluorescent siderophores in culture supernatant were performed at a wavelength of 400 nm, with each unit quantified as the concentration of siderophores that caused a 0.1 increase in optical density at *A*_400_. CAS assay method [48] was applied to determine the total siderophores released by isolates. Three loops from active colonies on cetrimide agar were added into 25 mL of Kings B broth and cultured at 25 °C for 48 h at 120 rpm. The fermented broth was centrifuged at 5000 rpm for 20 min. Specifically, 1.0 mL of supernatant was vortexed with 1.0 mL of CAS reagent, and the blank measurement was set by adding 1.0 mL of CAS reagent to 1.0 mL from an uncultured sterile medium. The conversion of blue into orange indicates the existence of siderophores. The reaction was assayed *A*_630_ nm after 20 min of incubation at room temperature.

### 4.5. Optimization of Siderophore Production

Nutritional factors greatly affect siderophore productivity. For this, the effect of common media such as Kings B, succinate, GASN, and glucose medium was assessed at pH 6.0. Kings B medium comprised (g/L) K_2_HPO_4_ (1.5), peptone (20.0), MgSO_4_·7H_2_O (1.5), and glycerol (10.0 mL). Succinate medium consisted of (g/L) succinic acid (4.0), (NH_4_)_2_SO_4_ (1.0), K_2_HPO_4_ (6.0), MgSO_4_·7H_2_O (0.2), and KH_2_PO_4_ (3.0). The glucose medium consisted of (g/L) glucose (10.0), K_2_HPO_4_ (0.56), and urea (0.85). GASN medium composed of (g/L) glucose (7), L-asparagine monohydrate (2), Na_2_HPO_4_ (0.96), KH_2_PO_4_ (0.44), and MgSO_4_·7H_2_O (0.2) [17]. The cultures were incubated at 25 °C with a shaking of 120 rpm for 48 h. Un-inoculated media were worked as control. The selected medium was supplemented with organic acids at 0.4% (*w*/*v*). The effect of physical conditions, such as the incubation period, on productivity was studied by growing the tested isolate in the production medium and incubating for different periods (0–96 h). The effect of pH was studied by growing the selected isolate in the production medium at initial pHs adjusted to 5.0–10.0. The influence of incubation temperature on siderophore biosynthesis was tested at 15–60 °C under the standard optimized conditions. At the end of each test, siderophore production was measured following the standard procedures mentioned above.

### 4.6. Purification of Siderophore

Growing cells were separated from the growth medium by centrifugation at 5000 rpm for 20 min, and the macromolecules were removed by passing through a 0.2 µm membrane and then adjusted to pH 6.0 with 1 N HCl. The PVD was then partially refined by mixing the filtrate with chloroform at a 1:1 (*v*/*v*) ratio in a separating funnel with frequent shaking for 10 min. The upper green PVD/chloroform layer was separated from the top medium layer. Evaporation of solvent was performed by a Hei-VAP rotary evaporator (Heidolph Instruments, Schwabach, Germany). The recovered PVD was dissolved in deionized water at 10 mg/mL concentration. The suspension was applied onto an Amberlite XAD-4 column (2.5 × 20 cm^2^) that was prepared by suspending the resin in distilled water at 20 g/L concentration and left for soaking at 4 °C for 6 h. The column was washed with 100 mL of deionized water to allow the binding of PVD with resin and the migration of undesirable free materials. The adsorbed PVD was then eluted with 50% (*v*/*v*) methanol at a speed of 5 mL/min, and 2 mL fractions were then collected. The siderophore-containing fractions of the major peak were pooled, and the solvent was evaporated. The recovered PVD was assayed for purity by a Reverse-Phase High-Performance Liquid Chromatography (RP-HPLC) (4.6 mm × 100 mm C18 column, Agilent Technologies Inc., Santa Clara, CA, USA) with a mobile phase consisting of two solvents; solvent A (0.1% formic acid) and solvent B (99.9% acetonitrile and 0.1% formic acid). The retention time (RT) was then determined [49]. For UV spectral scanning, the final purified PVD was resuspended in deionized water at 10 mg/mL concentration and adjusted to pH 7.0 with 1 N HCl/NaOH. After UV scanning of the neutral alkaline form, it was acidified to pH 3.0 using the lowest amount of 1 N HCl to scan the acidic form of PVD. UV analysis was performed with a UV-Vis spectrophotometer (Shimadzu UV-1900, Duisburg, Germany) at 300–900 nm, and the maximum absorbances were determined [50].

### 4.7. Thermal Stability and pH Stability of Siderophore

Siderophore preparations were thermally treated at 50–100 °C for 60 min. At 15 min intervals, small fractions were collected to quantify the residual siderophore at *A*_400_ and to scan at 300–700 nm for a shift in the maximum absorption peak. For pH stability assessment, siderophore preparations were adjusted to different pH values in the range of 5.0–9.5. In addition, CAS agars were adjusted to different pHs, the same as the siderophore with the lowest volume of NaOH/HCl system. Ten-millimeter well diameters were made in the center of Petri dishes with a sterile cork borer, and each was supplied with 0.5 mL of the siderophore preparation. Incubation was conducted at 35 °C for 24 h. The appearance of orange halos on CAS agars and their fluorescence under UV illumination was taken as an indication of their remaining activity and stability at the tested pH.

### 4.8. Anticandidal Activity of Siderophore

The initial inhibitory zones in Mueller–Hinton agar (MHA) plated with *C. auris* ATCC MYA-5001, *C. albicans* ATCC 14053, *C. glabrata* ATCC 15126, *C. tropicalis* ATCC 13803, and *C. parapsilosis* ATCC 22019 confirmed the anticandidal activity. Selected yeasts (1.0 × 10^8^ colony forming units/mL) were swabbed on the agar layer after settling. Next, 5 mm filter paper disks were loaded onto the agar surface, with each disk containing 50 µg of the tested siderophore in a 5 µL volume. Incubation was allowed for 24 h at 37 °C. The MICs were measured by the broth dilution method [51]. Dilute material to 128 µg/mL in sterile Brain Heart Infusion (BHI) Broth. Dilutions of 64 µg/mL to 1 µg/mL were prepared by two-fold dilution. Separate tubes received 10^8^ colony-forming units/mL yeast suspensions. A broth culture with no bacterial growth due to the exposure to 1 µg/mL amphotericin B served as the negative control (0% growth), while a broth tube not involving any inhibitory agents served as the positive control (100% growth). The incubation lasted 24 h at 37 °C with 120 rpm shaking. The MIC was identified as the mean of the lowest siderophore concentration that stopped microbe growth and the highest that permitted them to multiply. To determine the MFC, 100 µL from tubes above the MIC values were plated on MHA. MFC levels were insufficient for colony development. Microorganisms’ siderophore tolerance was calculated using the principle: tolerance = MFC/MIC. A tolerance of 16 or more makes siderophore static, while 4 or below makes it lethal [30].

### 4.9. Intracellular Iron Assay

The subinhibitory concentrations from the previous experiment prepared from 1.0–8.0 μg/mL of siderophore were used for the assessment of intracellular iron concentration and yeast growth (*A*_600_). The blank sample represented yeast growth in the absence of siderophore. Yeast cells were collected and then cleaned two times in PBS (pH 7.4). Acid digestion for harvested cells was carried out. Exactly 500 mg of biomass from each sample was incubated for 12 h in 2 mL of nitric acid mixed with 300 µL of deionized water. Preparations were then boiled at 105 °C for 2.5 h. One milliliter of 35% hydrochloric acid was added after the preparations were cooled down and reboiled in the same condition. After cooling, 300 µL of 30% hydrogen peroxide solution was supplemented and reboiled at the same condition. Final preparations were centrifuged at 5000 rpm for 3 min and then diluted in 200 mM acetic acid and 200 mM sodium acetate buffer. Iron ions were quantified by PerkinElmer AAnalyst 800 atomic absorption spectrometry (SpectraLab Scientific Inc., Alexandria, VA, USA) using (NH_4_)_2_Fe(SO_4_)_2_·6H_2_O as a standard.

### 4.10. Oxidative Stress Assays

Antioxidative enzymes and other whole-cell proteins were extracted from siderophore-treated cells by mixing 100 mg of lyophilized cells with urea lysis buffer consisting of 0.7 M β-mercaptoethanol, 1 mM EDTA, 1% SDS, 7 M urea, and 25 mM Tris HCl. The cell suspensions were exposed to nine cycles of 30 s homogenization to disrupt the cells. The cellular slurry was then centrifuged at 4 °C for 40 min at 13,500× *g* to precipitate the cell membrane for the assay of MDA due to peroxidation of membrane lipids. The resulting supernatant was utilized for assays of antioxidative enzymes and total protein concentrations (*A*_280_). Initially, the SOD enzymatic assay was performed. One hundred microliters were added to three milliliters of reaction mixture consisting of 0.2 M methionine, 30 mM EDTA, 1.5 M sodium carbonate, 60 μM Riboflavin, 2.25 mM nitroblue tetrazolium chloride, and 100 mM phosphate buffer and incubated at 25 °C for 10 min. SOD activity was expressed as U/mg protein after measuring *A*_560_ [52]. The unit (U) of SOD activity was identified as the quantity of enzyme causing a 50% reduction in inhibition of substrate.

In addition, a CAT assay was performed by adding fifty microliters of cell lysate with 1.5 mL of PBS (pH 7.4) and 950 μL of deionized water. The reaction was initiated by the addition of 500 µL of hydrogen peroxide solution prepared by mixing 775 μL of 30% hydrogen peroxide with 100 mL of deionized water. The decrease in measurable optical density at wavelength 240 nm of hydrogen peroxide was taken in the existence of the enzyme. The activity of CAT was defined as U/min/mg protein [52]. The activity of GST was evaluated using a protocol applied earlier [53]. The cell lysate was supplemented with 0.1 mM glutathione, 0.1 mM CDNB, and 50 mM PBS (pH 6.5), then absorbance was measured after 3 min at *A*_340_. GST activity was expressed as µM glutathione conjugated per minute after measuring *A*_340_. Lipid peroxidation assay in the form of MDA was performed by homogenization of 0.5 g yeast cells biomass with 0.1% (*w*/*v*) trichloroacetic acid and centrifuged at 21,000× *g* for 15 min. Four milliliters of 0.5% (*w*/*v*) thiobarbituric acid in 20% trichloroacetic acid (*v*/*v*) was mixed with 1 mL of cell lysate and boiled at 95 °C for 30 min. After cooling, centrifugation was performed at 150× *g* for 10 min. The optical density of supernatant was measured at wavelengths 532 nm and 600 nm [52].

### 4.11. SEM Study

The structural effect in both *C. albicans* ATCC 14053 and *C. tropicalis* ATCC 13803 after exposure to siderophore was examined under SEM. YPD broth was selected for the growth of tested species. It consisted of (g/L) peptone (20), dextrose sugar (20 g/L), and yeast extract (10) with a pH adjusted to 7.0. Two loopfuls from the active growth of *Candida* spp. were added to 25 mL medium, and the siderophore was supplemented at 10 µg/mL concentration. For blanks, the siderophore preparation was replaced with distilled deionized water. To develop cells, incubation was conducted at 37 °C at 120 rpm for 24 h. Cells were then collected by centrifugation at 5000 rpm for 15 min and fixed in 10% paraformaldehyde for 24 h. Cells were dehydrated in increasing ethanol concentrations (10, 30, 50, 70, 90%, and 99%). Alcohol exposure was 10 min except for pure ethanol, which allowed for 60 min. Cells were dried by lyophilization and then sputter-coated with chromium for 15 min. Tescan Vega3 SEM (Tescan, Brno, Czech Republic) was used at lower voltages to inspect the surface morphology of treated *Candida* spp.

### 4.12. Statistical Analysis

Unless otherwise mentioned, all readings were made from three biological replicates where the organism was allowed to grow under the same conditions three times, and the ultimate readings were presented as averages ± standard deviations. The statistical significance of differences between experimental groups was determined through one-way ANOVA and two-way ANOVA conducted by Excel 10 software. Differences were considered statistically significant when the *p*-value < 0.05.

## 5. Conclusions

The anticandidal activity of such siderophores may be considered a secondary function in the environment besides iron and mineral acquisition; they can inhibit and/or kill surrounding microorganisms by hyperaccumulation of iron inside the cells with a subsequent increase in the intracellular antioxidative enzymes SOD and CAT, and MDA due to membrane peroxidation. For defense, treated *Candida* produced protective exopolysaccharides and/or proteins. However, the high imbalance in homeostasis eventually resulted in cellular malformation and lysis after exposure. For this, the application of PVD-Mgrv7 siderophore in the suppression of *Candida* spp. may be a promising approach added to the anti-infective approaches that are less likely to induce resistance because it indirectly kills the microbial cells by hyperaccumulating iron rather than directly attacking the cell targets. The siderophore of *P. aeruginosa* Mgrv7 may be a useful agent to eradicate waterborne *Candida* and/or an active ingredient in topical ointments to suppress the skin-infecting *Candida*. Future research should focus on their antifungal activity against phytopathogenic fungi and their role in improving iron uptake by plants. Furthermore, their combination with antibiotics should be assessed.

## Figures and Tables

**Table 1:** Effect of Mgrv7 siderophore on the growth of various *Candida* species.

Tested Species	MIC (µg/mL)	MFC (µg/mL)	Tolerance Level
*Candida auris* ATCC MYA-5001	64	128	2.0
*Candida albicans* ATCC 14053	16	16	1.0
*Candida glabrata* ATCC 15126	128	>128	>1.0
*Candida tropicalis* ATCC13803	16	32	2.0
*Candida parapsilosis* ATCC 22019	32	64	2.0

## Data Availability

The datasets in this research can be found in online repositories. The names of the repositories and accession numbers are included in the article.
